# Inhibiting KDM6A Demethylase Represses Long Non-Coding RNA Hotairm1 Transcription in MDSC During Sepsis

**DOI:** 10.3389/fimmu.2022.823660

**Published:** 2022-01-28

**Authors:** Isatou Bah, Dima Youssef, Zhi Q. Yao, Charles E. McCall, Mohamed El Gazzar

**Affiliations:** ^1^ Department of Internal Medicine, East Tennessee State University College of Medicine, Johnson City, TN, United States; ^2^ Department of Internal Medicine, Section of Molecular Medicine, Wake Forest University School of Medicine, Winston-Salem, NC, United States

**Keywords:** sepsis, KDM6A, Hotairm1, MDSC, immune suppression

## Abstract

Myeloid-derived suppressor cells (MDSCs) prolong sepsis by promoting immunosuppression. We reported that sepsis MDSC development requires long non-coding RNA Hotairm1 interactions with S100A9. Using a mouse model that simulates the immunobiology of sepsis, we find that histone demethylase KDM6A promotes Hotairm1 transcription by demethylating transcription repression H3K27me3 histone mark. We show that chemical targeting of KDM6A by GSK-J4 represses Hotairm1 transcription, which coincides with decreases in transcription activation H3K4me3 histone mark and transcription factor PU.1 binding to the Hotairm1 promoter. We further show that immunosuppressive IL-10 cytokine promotes KDM6A binding at the Hotairm1 promoter. IL-10 knockdown repletes H3K27me3 and reduces Hotairm1 transcription. GSK-J4 treatment also relocalizes nuclear S100A9 protein to the cytosol. To support translation to human sepsis, we demonstrate that inhibiting H3K27me3 demethylation by KDM6A ex vivo in MDSCs from patients with protracted sepsis decreases Hotairm1 transcription. These findings suggest that epigenetic targeting of MDSCs in human sepsis might resolve post-sepsis immunosuppression and improve sepsis survival.

## Introduction

Sepsis is a life-threatening organ dysfunction caused by a dysregulated host response to infection and a leading cause of death and critical illness (1, 2). Sepsis may enable a profound innate and adaptive immune suppression following its acute hyperinflammatory response (3–5). The immunosuppressive state causes CD4 and CD8 T cell exhaustion (3, 6), incites new infections and reactivates latent viruses (7, 8), increases the incidence of chronic critical illness (9), and raises morbidity and mortality rates (3, 9). There are no molecular-targeted sepsis treatments.

Myeloid-derived suppressor cells (MDSCs) are pathologically activated neutrophils and monocytes that emerge from myeloid progenitors during aberrant stimulation of myeloid progenitors/precursors with cytokines and growth factors, and which enter sites of infection and inflammation (10–12). MDSCs continue to increase in blood, spleen, and bone marrow in mice and humans with sepsis, contributing to the protracted sepsis phenotype (13–16), called persistent inflammation, immunosuppression, and catabolism syndrome (PICS) (9, 15). We previously reported that MDSCs generation in a clinically relevant mouse sepsis model and in septic humans is supported by nuclear accumulation of inflammatory protein, S100A9 (17). Mice deficient in S100A9 fail to generate MDSCs and protracted sepsis (17). Accordingly, nuclear S100A9 protein promotes immunosuppressive MDSCs (17). We also reported that levels of lncRNA Hotairm1 in MDSCs increase in mice with later/protracted sepsis (18). Hotairm1 transfers cytosolic S100A9 to the nucleus in MDSCs, at least in part, to drive MDSC immunosuppressive effects.

Epigenetic mechanisms maintain homeostasis, as well as increase and decrease in gene transcription [reviewed in (19)]. Infection-induced epigenetic changes in innate immunity cells can prolong immunosuppression during later/chronic sepsis (20–22). Immunosuppression associated with later sepsis leads to secondary infections and mounting mortality (3, 20, 23). Using the polymicrobial mouse model of sepsis, we discovered an epigenetic signature directed by the histone lysine demethylase, KDM6A, in MDSCs from mice with later sepsis (24). In this study, we extended our mechanistic and translational research by identifying KDM6A as a promoter of the epigenetic exchanges that induce Hotairm1 transcription in MDSCs during sepsis.

KDM6A, also known as UTX, belongs to the H3K27me3-specific demethylase subfamily, KDM6. KDM6 demethylases contain a JmjC catalytic domain that demethylates tri-methylated Lys 27 on histone H3 (H3K27me3) to promote gene activation (25–27). Depletion of KDM6A also increases H3K27me3 to silences HOX genes during myeloid cell differentiation (25, 28). Targeting KDM6A reduces inflammatory responses in human macrophages (29). KDM6A binding to the Hotairm1 promoter in MDSCs from mice with later sepsis correlates with Hotairm1 transcription activation (24).

Here, we found that KDM6A specific inhibitor GSK-J4 supports an exchange between H3K27me3 and H3K4me3 at Hotairm1 promoter in mouse and human MDSCs, thereby mediating the effect of other bioactive mediators of sepsis immunosuppression (e.g., IL-10).

## Materials and Methods

### Mice

Male C57BL/6 mice (8 to 10 weeks old) were purchased from the Jackson Laboratory (Bar Harbor, ME) and housed in a pathogen-free facility. All experiments were conducted according to National Institutes of Health guidelines and were approved by the East Tennessee State University Animal Care and Use Committee.

Several clinical and experimental studies have shown that cell-mediated immune responses are depressed in males while enhanced in females during sepsis (30, 31) and that the female mouse is more immunologically competent than the male mouse in surviving CLP insult (32). And because MDSCs suppress both innate and adaptive immune responses, we used male mice to assess the maximal effect of this immunosuppressive cell population on sepsis outcome.

### Induction of Sepsis

Polymicrobial sepsis was induced by cecal ligation and puncture (CLP) as described previously (33). Briefly, a midline abdominal incision was made, and the cecum was ligated distal to the ileocecal valve and punctured twice with a 23-gauge needle. A small amount of feces was extruded into the abdominal cavity. Mice received (i.p.) 1 ml lactated Ringers plus 5% dextrose for fluid resuscitation. To establish intra-abdominal infection and approximate the clinical condition of human sepsis (34) and delay in MDSC development, mice were subcutaneously administered antibiotic (imipenem; 25 mg/kg body weight) in 0.9% saline at 8 and 16 h after CLP. These manipulations result in acute sepsis and post-sepsis phases, with high mortality (~60-70%) during the post-sepsis phase (13). Survival was followed for 28 days. Mice moribund during early sepsis (defined as the first 5 days after CLP) or later sepsis (day 7-28) (33) were euthanized and analyzed. Mice were considered moribund if they experienced hypothermia (< 34°C) or loss of righting reflex. A corresponding number of mice from the control/sham group were also analyzed at the same time point.

### Patients

Patients 18 years or older admitted to Johnson City Medical Center and Franklin Woods Community Hospital in Johnson City, Tennessee with sepsis or septic shock participated in the study. Sepsis patients were identified based on the presence of documented or suspected infection along with an acute increase of ≥2 points in SOFA (sequential organ failure assessment) score (1). The baseline SOFA score is assumed to be zero in patients without preexisting organ dysfunctions, which are determined by PaO2, platelets count, Glasgow Coma Scale score, creatinine, and bilirubin levels. Septic shock patients presented with persistent hypotension requiring vasopressors to maintain MAP ≥65 mm Hg and had serum lactate >2 mmol/L despite adequate fluid resuscitation (1). Patients presented with infections related to gram-negative or gram-positive bacteria, and the primary sites of infection included the urinary tract, circulation, and respiratory tract. Patients had at least 1 comorbid condition, such as nephropathy, psoriasis, splenectomy, colon cancer, or pulmonary disease. Patients with leukopenia due to chemotherapy or with glucocorticoid therapy or HIV infection were excluded. Patients were in two categories: early sepsis and later sepsis (i.e., protracted sepsis), based on time of diagnosis. The early sepsis group within 1-5 days of sepsis diagnosis and later sepsis more than 6 days. Later sepsis blood procurement occurred at days 6-68 after sepsis diagnosis. Blood samples obtained from healthy control subjects were supplied by BioIVT (Gray, TN). A total of 18 sepsis patients and 10 healthy subjects are included in this study. The study was approved by the Institutional Review Board (IRB) of the East Tennessee State University (IRB#: 0714.6s). Signed informed consent was obtained from all participants.

### Myeloid-Derived Suppressor Cells (MDSCs)

Gr1^+^CD11b^+^ MDSCs were isolated from mouse bone marrow by negative selection using EasySep mouse MDSC isolation kit (Cat# 19867) according to the manufacturer’s protocol (Stemcell Technologies, Cambridge, MA). Briefly, the bone marrow was flushed out of the femurs with RPMI-1640 medium under aseptic conditions. A single-cell suspension was made by filtering through a 70 μm mesh nylon strainer, followed by incubation with erythrocyte lysis buffer and washing. The cell suspension was incubated with a biotin-coupled antibody cocktail (binds to all non-Gr1^+^/CD11b^+^ cells) at room temperature for 10 min, followed by adding streptavidin-coated magnetic particles and incubating at room temperature for 5 min. Sample tubes were placed into a magnet for 3 min, and the enriched (flow-through) cell suspension containing Gr1^+^CD11b^+^ cells was transferred to a fresh tube. The cells were more than 90% Gr1^+^ and CD11b^+^ positive as determined by flow cytometry. For cell culture, the Gr1^+^CD11b^+^ cells were incubated with RPMI-1640 medium (Invitrogen, Carlsbad, CA) supplemented with 100 U/ml penicillin, 100 μg/ml streptomycin, 2 mM L-glutamine (HyClone Laboratories, Logan, UT), and 10% fetal bovine serum (Atlanta Biologicals, Lawrenceville, GA) at 37°C and 5% CO_2_.

For human MDSCs, peripheral blood mononuclear cells were isolated from whole blood by density gradient centrifugation using Ficoll-Paque Plus according to the manufacturer’s protocol (GE Healthcare Life Sciences, Marlborough, MA), and then depleted of HLA-DR^+^ cells *via* positive selection using a biotin-coupled anti-HLA-DR antibody (Cat# 13-9956-82, eBioscience, San Diego, CA) and anti-biotin microbeads (Miltenyi Biotec, Auburn, CA). Next, the remaining cells were positively selected using biotin-coupled anti-CD33 (Cat# MA1-19522, Invitrogen) and anti-LOX-1 antibodies (Cat# 130-122-119) (Milteny Biotec).

For inhibition of KDM6A demethylase activity, GSK-J4 HCl (Cat# S7070, Selleckchem, Houston, TX) was reconstituted in DMSO at variable concentrations. A 0.1% DMSO was used as a control.

### Knockdown of IL-10

Inhibition of IL-10 in Gr1^+^CD11b^+^ cells was performed using pools of gene-specific or scrambled (control) siRNAs (Santa Cruz Biotechnology, Dallas, TX). The siRNA mixture was suspended in HiPerFect reagent (Qiagen, Germantown, MD) at a 0.5 μM final concentration. The cells were transfected and incubated with RPMI-1640 medium for 36 h.

### Chromatin Immunoprecipitation (ChIP)

ChIP was performed using ChIP-IT Express Enzymatic Shearing kit according to the manufacturer’s protocol (Active Motif, Carlsbad, CA). Briefly, mouse Gr1^+^CD11b^+^ cells and human CD84^+^LOX1^+^HLA-DR^-^ cells were isolated and fixed in 1% formaldehyde in a minimal culture medium at room temperature for 10 min, to cross-link DNA-protein complexes. After washing, cells were resuspended in lysis buffer containing protease inhibitor cocktail and incubated on ice for 1 h. The cell lysate was cleared by centrifugation at 5,000 rpm and 4°C for 10 min. The pelleted nuclei were then resuspended in digestion buffer containing an enzymatic shearing cocktail and incubated at 37°C for 10 min. The sheared chromatin (supernatant) was recovered by centrifugation at 15,000 rpm for 10 min at 4°C. Ten microliters of the chromatin solution was reserved as an “input DNA” sample. Next, 5 μg of antibody against KDM6A (Cat# 33510S, Cell Signaling Technology), PU.1 (Cat# MA5-15064, Invitrogen), H3K4me3 (Cat# MBS9401981), H3K27me3 (Cat# MBS3010330) (MyBioSource, San Diego, CA), Ezh2/ENX-1 (Cat# 5246S, Santa Cruz Biotechnology), or isotype control antibody and 25 μl of protein G-coated magnetic beads were added to 150 μl of the sheared chromatin, and the chromatin was then immunoprecipitated at 4°C overnight with rotation. The chromatin/antibody complexes captured on the beads were washed three times in ChIP buffer and eluted by incubation for 15 min in 50 μl elution buffer. The DNA-protein cross-links were reversed by adding 50 μl of reverse cross-linking buffer to the eluted chromatin and the samples were incubated, along with the “input” DNA, at 95°C for 15 min. After treatment with 5 μl of proteinase K at 37°C for 1 h (to degrade proteins), the ChIPed DNA was recovered and stored at -20°C until analyzed by PCR as described below.

### Quantitative Real-Time PCR

For ChIP analysis, the ChIPed DNA was amplified by real-time qPCR using QuantiTect SYBR Green PCR Master Mix (Qiagen) and primers designed to amplify a 220 bp fragment that contains a PU.1 binding site at -199 to -194 of the mouse Hotairm1 promoter. These primers were: PU.1 forward 5’-tcccagagtcgccactgccaa-3’; PU.1 reverse 5’-tagagtcacgtgtcctcccc-3’ (Integrated DNA Technologies, Coralville, IA). For human Hotairm1, we used primers designed to amplify a 220 bp fragment that contains a PU.1 binding site at -195 to -200 (lower strand) of the human Hotairm1 promoter. These primers were: PU.1 forward 5’-gtatggggtattccaggaagg-3’; PU.1 reverse 5’-gaggctcagccattggctga-3’. The PCR reactions were performed in duplicate in 50 μl volumes. The cycling conditions were as follows: 1 cycle at 95°C for 15 min, 35 cycles at 94°C, 58°C, and 72°C for 30 s each, and a final cycle at 72°C for 10 min.

For the analysis of Hotairm1 expression, total RNA was isolated using TRIzol reagent (Invitrogen) and subjected to reverse transcription using QuantiTect Reverse Transcription kit (Qiagen). The cDNA was amplified by real-time qPCR using QuantiTect SYBR Green PCR Master Mix kit and pre-designed qPCR Primer Assays specific to mouse Hotairm1 (assay ID# LPM17359A) and GAPDH (assay ID# QT01658692), and human Hotairm1 (assay ID# LPH10483A) and GAPDH (assay ID# QT00079247) (all from Qiagen). The expression level was calculated using the 2^-ΔΔCt^ cycle threshold method. The values were normalized to input DNA or GAPDH RNA level, and the results are presented as a fold change relative to the control samples.

### Western Blot

Whole-cell lysates were prepared using 1x RIPA lysis buffer containing 50 mM Tris-HCl [pH 7.4], 150 mM NaCl, 1% NP-40, 0.25% sodium deoxycholic acid, 1 mM EDTA (Millipore, Temecula, CA), and 1x protease inhibitor cocktail. Nuclear and cytoplasmic extracts were prepared using the NE-PER nuclear and cytoplasmic extraction kit according to the manufacturer’s protocol (Pierce, Rockford, IL). Protein extracts were resolved onto SDS-10% polyacrylamide gel (Bio-Rad, Hercules, CA) and transferred to nitrocellulose membranes (Thermo Fisher Scientific, Waltham, MA). The membranes were blocked with 5% milk in Tris-buffered saline/Tween-20 for 1 h at room temperature and then probed overnight at 4°C with an antibody specific to PU.1 (Cat# MA5-15064, Invitrogen, Carlsbad, CA), Ezh2 (Cat# 166609), IL-10 (Cat# sc-32815), S100A9 (Cat# sc-58706) (Santa Cruz Biotechnology), KDM6A (Cat# 33510S, Cell Signaling Technology, Danvers, MA) antibody. After washing, blots were incubated with the appropriate HRP-conjugated secondary antibody for 2 h at room temperature. Proteins were detected with the enhanced chemiluminescence detection system (Thermo Fisher Scientific), the bands were visualized using the ChemiDoc XRS System (Bio-Rad), and the images were captured with the Image Lab Software V3.0. The membranes were stripped and reprobed for β-actin (Invitrogen).

### Statistical Analysis

Data were analyzed with Microsoft Excel, V3.0. Values are expressed as mean ± SD. Differences between two groups were determined by a two-tailed student’s *t*-test. *P*-values of < 0.05 were considered statistically significant.

## Results

### Inhibiting KDM6A Demethylase Activity by GSK-J4 Increases H3K27me3 Levels on the Hotairm1 Promoter

Our previous studies showed that Hotairm1 modifies the S100A9 protein’s function to generate MDSCs during later sepsis (17, 18). Hotairm1 expression in MDSCs is induced by the binding of transcription factor PU.1 to its proximal promoter. This process is facilitated by an exchange of the transcriptional repressive histone mark H3K27me3 for the permissive mark H3K4me3 as sepsis progresses to the later, protracted state (24). KDM6A is a selective demethylase of H3K27me3, and targeting KDM6A activity *in vivo* in mice (35) and in human macrophages (29) increases the H3K27me3 level on promoters of target genes. Here, we used KDM6A potent and specific inhibitor GSK-J4 to modulate Hotairm1 RNA transcription in sepsis MDSCs.

**Figure 1:**
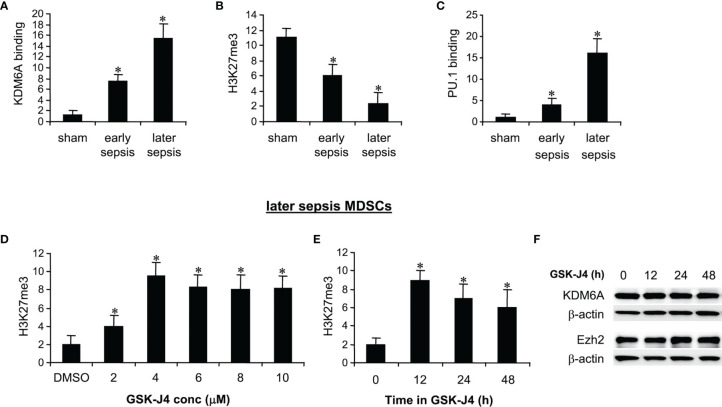
Inhibition of KDM6A in MDSCs increases H3K27me3 on the Hotairm1 promoter. **(A)** Gr1^+^CD11b^+^ cells were purified from bone marrow cells by negative selection using magnetic beads and anti-Gr1 and anti-CD11b antibodies. **(A–C)** Chromatin immunoprecipitation (ChIP) assay to detect KDM6A, H3K27me3, and PU.1 at Hotairm1 promoter. Chromatin was immunoprecipitated with anti-KDM6A, anti-H3K27me3, anti-PU.1, or anti-IgG control antibody. The ChIPed DNA was analyzed by real-time qPCR. Samples values were normalized to the “input” DNA (DNA isolated before the immunoprecipitation) and are presented relative to the IgG-immunoprecipitated samples (set at 1-fold). **(D)** Dose-dependent inhibition of KDM6A. The Gr1^+^CD11b^+^ cells were incubated with varying concentrations of KDM6A inhibitor GSK-J4 in DMSO or 0.1% DMSO alone for 12 h. **(E)** Time-dependent inhibition of KDM6A. The cells were incubated with 4 μM of GSK-J4 for the indicated times. Chromatin was immunoprecipitated with anti-H3K27me3 antibody. Data represent mean ± SD for 5-6 mice per group, pooled from independent experiments. *p < 0.05, early or later sepsis vs. sham **(A–C)**; *p < 0.05, vs. DMSO **(D)**; *p < 0.05, vs. 0 h **(E)**. **(F)** Western blotting of Ezh2 and KDM6A in Gr1^+^CD11b^+^ cells following GSK-J4 inhibition. The results are representative of two Western blots.

ChIP analysis of Hotairm1 promoter showed that KDM6A binding was significantly higher in MDSCs from mice with later sepsis compared with early sepsis. The increase in KDM6A binding was accompanied by a decrease in H3K27me3 and a subsequent increase in PU.1 binding and Hotairm1 transcription [**Figures 1A–C** and (24)]. We tested whether the inhibition of KDM6A demethylase activity can increase H3K27me3 deposition on the Hotairm1 promoter. MDSCs from mice with later sepsis, which exhibit the most decrease in H3K27me3 and increase in KDM6A binding, were incubated with DMSO or increasing concentrations of GSK-J4 for 12 h. As shown in **Figure 1D**, H3K27me3 deposit on Hotairm1 promoter significantly increased in the presence of 4 μM concentration of GSK-J4, and its level slightly decreased and plateaued at concentrations higher than 4 μM. There were no significant differences in cell death as determined by trypan blue staining within the 2-10 μM concentration range (data not shown).

We next used 4 μM concentration to determine the optimal incubation time for the GSK-J4 effect on KDM6A inhibition. MDSCs treated with GSK-J4 for 12 h exhibited the highest increase in H3K27me3 compared to 24 or 48 h (**Figure 1E**). In addition, treatment with 4 μM for 12-48 h did not affect the cellular levels of KDM6A (**Figure 1F**) or Ezh2 protein, which trimethylates H3K27 in MDSCs (24). Therefore, we used 4 μM concentration and 12 h incubation time in all subsequent experiments.

### Inhibiting KDM6A Activity Decreases H3K4me3 Levels on Hotairm1 Promoter in MDSCs

**Figure 2:**
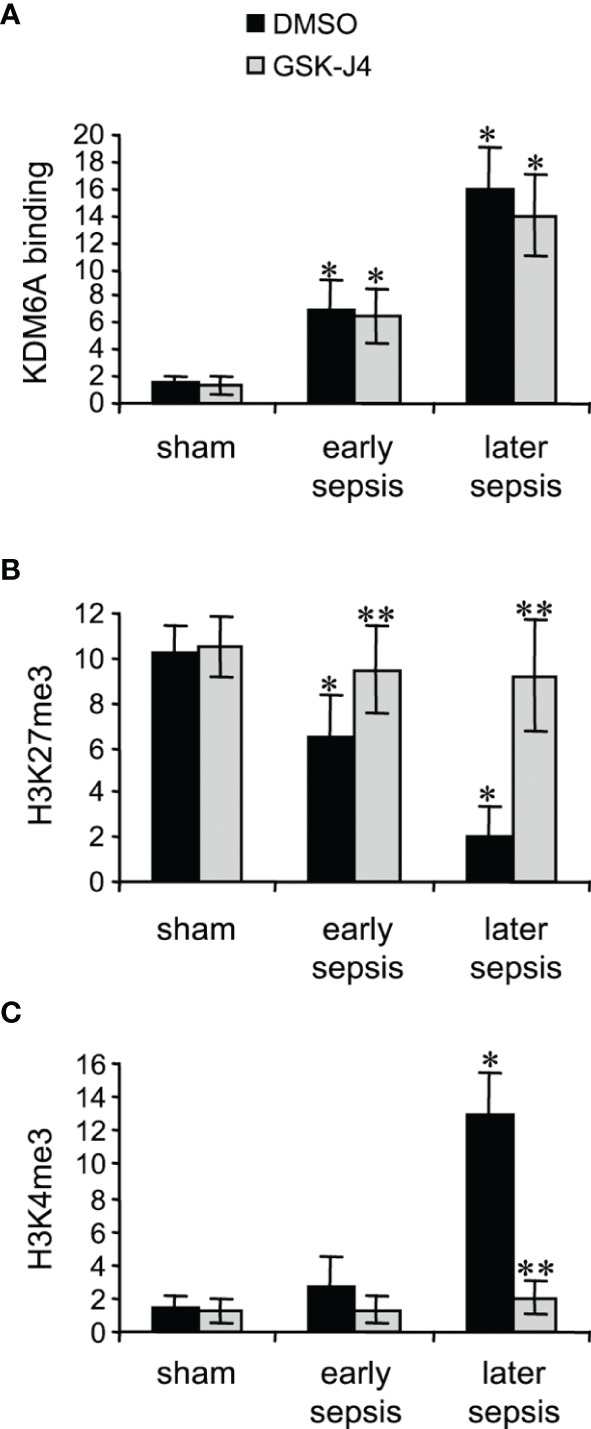
Inhibition of KDM6A in MDSCs decreases H3K4me3 on the Hotairm1 promoter. Gr1^+^CD11b^+^ cells were purified from bone marrow cells by negative selection using magnetic beads and anti-Gr1 and anti-CD11b antibodies. The cells were incubated with 4 μM of GSK-J4 or 0.1% DMSO for 12 h. Chromatin was isolated as described in **Figure 1** and then immunoprecipitated with anti-KDM6A **(A)**, anti-H3K27me3 **(B)**, anti-H3K4me3 **(C)**, or IgG control antibody. The ChIPed DNA was analyzed by qPCR as described in **Figure 1**. Data represent mean ± SD for 4-6 mice per group, pooled from independent experiments. *p < 0.05, early or later sepsis vs. sham; **p < 0.5, GSK-J4 vs. DMSO.

KDM6A protein binds to Hotairm1 proximal promoter following sepsis initiation, and its binding peaks in later sepsis MDSCs (24). KDM6A decreases H3K27me3 and simultaneously increases the transcriptional permissive mark H3K4me3 (24). Chromatin collected from MDSCs isolated from sham and septic mice was used to analyze levels of KDM6A, H3K27me3, and H3K4me3 following treatment with 4 μM GSK-J4 for 12 h, using ChIP assay. GSK-J4 treatment did not affect KDM6A binding at the Hotairm1 promoter (**Figure 2A**). Notably, inhibition of KDM6A demethylase activity markedly increased H3K27me3 and nearly diminished H3K4me3 deposit on the promoter (**Figures 2B, C**). These results support that GSK-J4 effectively inhibits KDM6A demethylase activity and increases the amount of H3K27me3 on the Hotairm1 promoter in MDSCs.

### Inhibiting KDM6A Activity Decreases PU.1 Binding and Transcription in MDSCs

**Figure 3:**
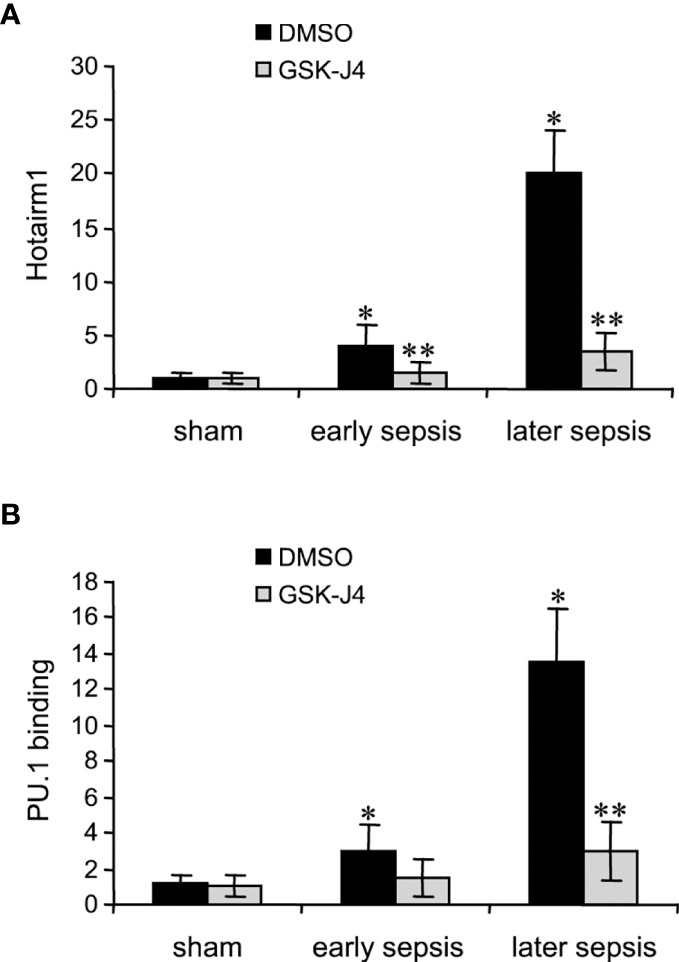
Inhibition of KDM6A in MDSCs reduces Hotairm1 transcription. Gr1^+^CD11b^+^ cells were purified from bone marrow cells by negative selection using magnetic beads and anti-Gr1 and anti-CD11b antibodies. The cells were incubated with 4 μM of GSK-J4 or 0.1% DMSO for 12 h. **(A)** Total RNA was isolated and Hotairm1 levels were determined by real-time RT-qPCR using qPCR Primer Assay specific to Hotairm1. Sample values were normalized to GAPDH RNA as an internal control and are presented relative to sham (1-fold). **(B)** KDM6A inhibition reduces PU.1 binding at the Hotairm1 promoter. Chromatin was isolated as described in **(A)** and immunoprecipitated with anti-PU.1 or anti-IgG control antibody. The ChIPed DNA was analyzed by qPCR as described in **(A)**. Data represent mean ± SD for 5-6 mice per group, pooled from independent experiments. Data in **(A)** are presented relative to sham and data in **(B)** are presented relative to the IgG-immunoprecipitated samples (1-fold). *p < 0.05, early or later sepsis vs. sham; **p < 0.05, GSK-J4 vs. DMSO.

Transcription of Hotairm1 RNA in MDSCs during sepsis is induced by PU.1 binding at its proximal promoter, which is dependent on the demethylation of H3K27me3 by KDM6A and the deposit of H3K4me3 (24). We next assessed the effect of KDM6A inhibition on PU.1 binding and Hotairm1 transcript levels. Hotairm1 levels were normalized to GAPDH. Note GAPDH expression is not stable during sepsis. GSK-J4 treatment for 12 h significantly reduced Hotairm1 transcripts, with a pattern that mirrored a decrease in PU.1 binding (**Figure 3**). These findings supported our hypothesis that removal of H3K27me3 from Hotairm1 promoter *via* KDM6A-mediated demethylation plays a major role in the induction of Hotairm1 in MDSCs during sepsis.

### IL-10 Induces KDM6A Binding at Hotairm1 Promoter in MDSCs

**Figure 4:**
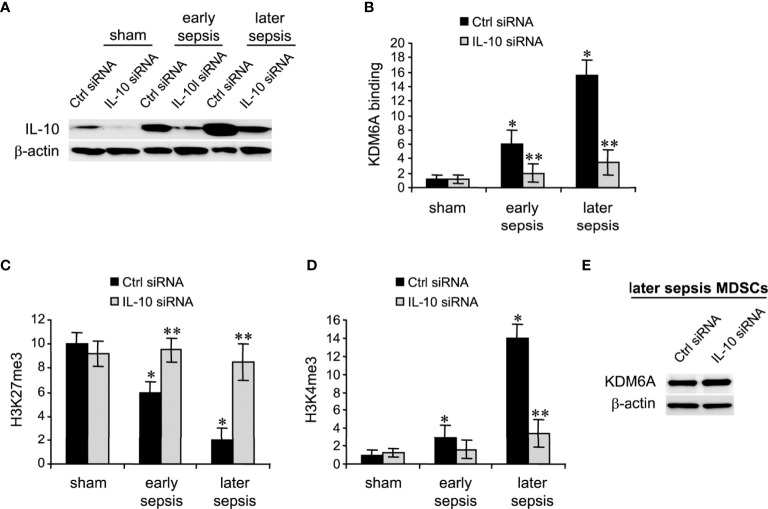
Knockdown of IL-10 in MDSCs inhibits KDM6A binding at the Hotairm1 promoter. Gr1^+^CD11b^+^ cells were purified from bone marrow cells by negative selection using magnetic beads and anti-Gr1 and anti-CD11b antibodies. The cells were transfected with IL-10-specific or scrambled/control siRNA for 36 h. **(A)** Western blotting of IL-10 following the knockdown. The results are representative of two Western blots. **(B–D)** Chromatin was isolated as described in **Figure 1** and then immunoprecipitated with anti-KDM6A, anti-H3K27me3, anti-H3K4me3, or anti-IgG control antibody. The ChIPed DNA was analyzed by qPCR using primers that amplify the promoter sequences surrounding PU.1 binding site. Samples values were normalized to the “input” DNA and are presented relative to the IgG-immunoprecipitated samples (1-fold). Data represent mean ± SD for 4-5 mice per group, pooled from independent experiments. *p < 0.05, early or later sepsis vs. sham; **p < 0.05, IL-10 siRNA vs. Ctrl siRNA. **(E)** Western blotting of KDM6A following IL-10 after the knockdown. The results are representative of two Western blots. Ctrl, control.

The immunosuppressive IL-10 cytokine, whose levels increase significantly during later sepsis, promotes the Hotairm1-mediated shuttling of S100A9 protein to the nucleus in MDSCs (36). We have recently shown that the programming of myeloid precursors into immunosuppressive MDSCs in later sepsis is dependent on IL-10 (18). We hypothesized that IL-10 induces this process by promoting KDM6A-mediated induction of Hotairm1. To test this hypothesis, we knocked down IL-10 in MDSCs and assessed KDM6A binding and H3K27me3 and H3K4me3 levels at Hotairm1 promoter using ChIP assay. Western blotting showed the knockdown was efficient in reducing IL-10 protein (**Figure 4A**). Notably, the binding of KDM6A at the Hotairm1 promoter was significantly reduced (**Figure 4B**). The decrease in KDM6A binding significantly increased H3K27me3 and decreased H3K4me3 levels on the Hotairm1 promoter (**Figures 4C, D**). These changes were not due to a decrease in KDM6A expression, because KDM6A protein level was not affected by IL-10 knockdown (**Figure 4E**).

**Figure 5:**
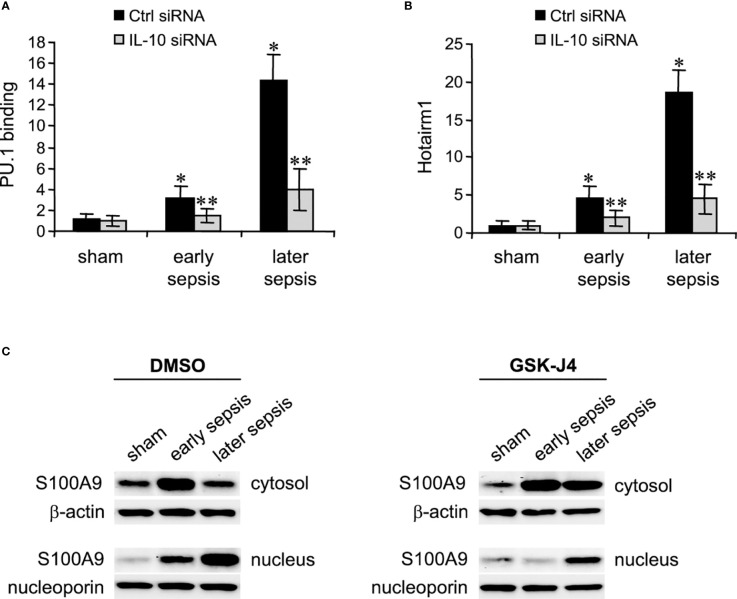
PU.1 binding and Hotairm1 transcripts in MDSCs after IL-10 Knockdown. Gr1^+^CD11b^+^ cells were purified from bone marrow cells and transfected with IL-10-specific or scrambled/control siRNA for 36 h. **(A)** PU.1 binding at Hotairm1 promoter. Chromatin was immunoprecipitated with anti-PU.1 or anti-IgG isotype control antibody. The ChIPed DNA was analyzed by qPCR. Samples values were normalized to the “input” DNA and are presented relative to the IgG-immunoprecipitated samples (1-fold). **(B)** Total RNA was isolated and Hotairm1 levels were determined by real-time RT-qPCR using qPCR Primer Assay specific to Hotairm1. Sample values were normalized to GAPDH RNA as an internal control and are presented relative to sham (1-fold). Data represent mean ± SD for 4-6 mice per group, pooled from independent experiments. *p < 0.05, early or later sepsis vs. sham; **p < 0.05, IL-10 siRNA vs. Ctrl siRNA. **(C)** Inhibition of KDM6A moves S100A9 protein from the nucleus to the cytosol. Western blot analysis of S100A9 protein localization. The cells were incubated with 4 μM of GSK-J4 or 0.1% DMSO for 12 h. Protein extracts were used to determine S100A9 levels in the cytosol and nucleus. The results are representative of two Western blots.

Next, we determined PU.1 binding and Hotairm1 transcript levels after IL-10 knockdown. As shown in **Figures 5A, B**, depletion of IL-10 resulted in significant decreases in PU.1 binding and Hotairm1 transcripts. These findings suggest that IL-10 induces the epigenetic changes that promote Hotairm1 transcription in MDSCs during sepsis.

### Inhibiting KDM6A Activity Relocalizes S100A9 to the Cytosol in MDSCs

Hotairm1 shuttles S100A9 protein to the nucleus in myeloid precursors to program them into MDSCs during later sepsis (18). Because the results described above showed that inhibition of KDM6A suppressed Hotairm1 transcription, we assessed the effect of KDM6A inhibition on S100A9 protein localization. MDSCs were treated with DMSO or GSK-J4 for 12 h, and nuclear and cytosolic extracts were analyzed by Western blotting. GSK-J4 treatment resulted in S100A9 relocalization to the cytosol in later MDSCs, similar to early sepsis MDSCs, which are not immunosuppressive (**Figure 5C**). These results suggest that induction of Hotairm1 due to H3K27me3 demethylation by KDM6A is pivotal in targeting S100A9 protein to the nucleus in later MDSCs.

### GSK-J4 Treatment in MDSCs From Sepsis Patients Inhibits KDM6A Demethylase Activity and Hotairm1 Transcription

**Figure 6:**
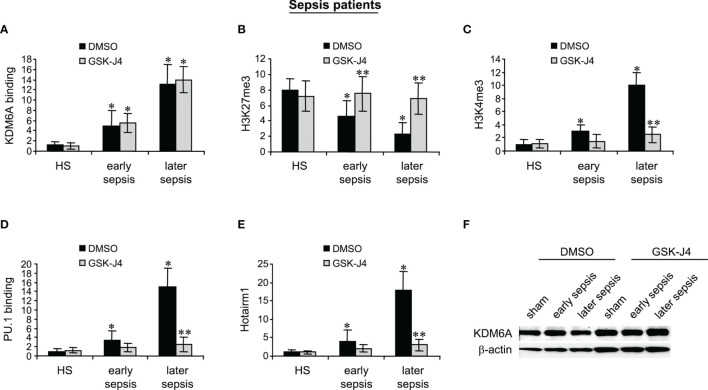
Inhibition of KDM6A attenuates PU.1 binding and Hotairm1 transcription in MDSCs from septic patients. PBMCs were first purified and depleted of the HLA-DR^+^ cells using biotin-coupled anti-HLA-DR antibody and anti-biotin microbeads, followed by the positive selection of CD33^+^LOX1^+^ cells with biotin-coupled anti-CD33 and anti-LOX1 antibodies. These CD33^+^LOX1^+^HLA-DR^-^ cells were incubated with 4 μM of GSK-J4 or 0.1% DMSO for 12 h. **(A–D)** Chromatin was isolated and immunoprecipitated with anti-KDM6A, anti-H3K27me3, anti-H3K4me3, anti-PU.1, or anti-IgG control antibody. The ChIPed DNA was analyzed by real-time qPCR. Samples values were normalized to the “input” DNA and are presented relative to the IgG-immunoprecipitated samples (1-fold). **(E)** Total RNA was isolated and Hotairm1 levels were determined by real-time RT-qPCR. Sample values were normalized to GAPDH RNA as an internal control and are presented relative to HS (1-fold). Data represent mean ± SD for 3-4 patients or HS per group. *p < 0.05, early or later sepsis vs. HS; **p < 0.05, GSK-J4 vs. DMSO. **(F)** Western blotting of KDM6A. The results are representative of two Western blots. HS, healthy subjects.

MDSCs were isolated from the peripheral blood from patients with early sepsis and those that developed a later, protracted sepsis state. The cells were treated with DMSO or GSK-J4 for 12 h. ChIP assays revealed that GSK-J4 treatment did not affect KDM6A binding at human Hotairm1 promoter (**Figure 6A**). Inhibition of KDM6A activity significantly increased H3K27me3 levels on the promoter, which resulted in a significant decrease in H3K4me3 levels (**Figures 6B, C**). Importantly, this increase in H3K27me3 prevented PU.1 binding and subsequent activation of Hotairm1 transcription, as demonstrated by the significant decrease in Hotairm1 transcripts (**Figures 6D, E**). In addition, Western blotting showed that GSK-J4 did not impact the cellular level of KDM6A protein (**Figure 6F**). These results support that GSK-J4 effectively inhibits KDM6A demethylase activity and targets epigenetic-mediated Hotairm1 transcription in human MDSCs during sepsis.

## Discussion

**Figure 7:**
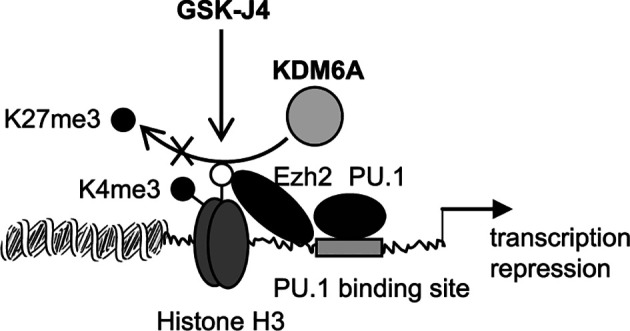
A diagram depicting the effect of GSK-J4 on Hotairm1 transcription in MDSCs. During sepsis, histone demethylase KDM6A binds at the Hotairm1 promoter around the PU.1 binding site and removes K27me3, which is deposited by histone methyltransferase Ezh2. The removal of K27me3 leads to the deposit of K4me3 and activation of Hotairm1 transcription. This process can be blocked with GSK-J4, which inhibits KDM6A demethylase activity and thus prevents the removal of K27me3, leading to transcription repression of Hotairm1.

The major finding of this study is that targeting KDM6A demethylase using GSK-J4 inhibits Hotairm1 transcription in mouse and human MDSCs. GSK-14 acts by inhibiting KDM6A demethylase activity (depicted in **Figure 7**) that catalyzes the removal of the methyl groups from tri-methylated histone lysine 27, H3K27me3 (29, 35). Mechanistically, KDM6A demethylates the transcription repressive H3K27me3 mark that is deposited on the Hotairm1 promoter as sepsis progresses to the later, protracted state. Furthermore, countering H3K27me3 promotes the transcription activation H3K4me3 mark implicated in Hotairm1 transcription. Notably, these epigenetic changes were also observed in MDSCs from spleens (**Supplementary Figure 1**), suggesting that epigenetic modifications at the Hotairm1 promoter are maintained systematically after MDSCs exit the bone marrow. Because Hotairm1 couples with S100A9 to program MDSCs during later sepsis, our findings underscore the importance of removing H3K4me3 in limiting excessive MDSC accumulation during later sepsis in humans.

GSK-J4 treatment for 12 h increased H3K27me3 levels without affecting cell viability. GSK-J4 targeting KDM6A is an effective therapeutic strategy against craniosynostosis (35), lymphoblastic and myeloid leukemias (37, 38), and breast, ovarian and prostate cancers (39–41). Kruidenier et al. (29) reported GSK-J4 as a specific inhibitor of KDM6A’s demethylase activity during macrophage inflammatory responses. Mechanistically, GSK-J4 prevents LPS-induced removal of H3K27me3 that is present at the TNFα transcription start site and inhibits TNFα expression in human primary macrophages (29). Our results showed that GSK-J4 treatment had no effects on KDM6A protein levels in MDSCs as reported by Kruidenier (29).

The trimethylation of H3K27 by zeste homolog 2 (Ezh2) is part of the epigenetic activity of the mammalian polycomb repressor complex PRC2 (42) and negatively regulates MDSCs in tumor-bearing mice (43). We reported that Ezh2 binds at Hotairm1 promoter in MDSCs with no change in binding levels before and during sepsis (24), but Ezh2 knockdown reduces H3K27me3 levels on Hotairm1 promoter (24). Although both Ezh2 and KDM6A protein expression in MDSCs remains unchanged during sepsis, KDM6A binding at Hotairm1 promoter increases significantly after sepsis induction. Of note, our results showed that GSK-J4 treatment does not affect either Ezh2 or KDM6A protein levels. We conclude that KDM6A plays an important role in reducing repressor mediator H3K27me3 on the Hotairm1 promoter, thereby increasing Hotairm1 during sepsis.

Removing H3K27me3 from the Hotairm1 promoter in MDSCs informed transcription activation by increasing the level of H3K4me3 deposition. H3K4Me3 commonly activates gene promoters (44, 45), including some lncRNAs (46, 47).

Immunosuppressive IL-10 cytokine commonly supports sepsis-induced immunosuppression (48) and its levels increase in mouse MDSCs as sepsis progresses to the later, protracted stage (13). IL-10 supports S100A9 protein translocation to the nucleus in MDSCs (36), concomitant with Hotairm1 binding to S100A9 (18). In this study, we used GSK-J4 treatment to relocate S100A9 to the cytosol in later sepsis MDSCs. By targeting KDM6A demethylase activity, S100A9 nuclear accumulation decreased in MDSCs, supporting the report that mice deficient in S100A9 do not generate MDSCs during sepsis (17). IL-10 knockdown in this study inhibited KDM6A binding at Hotairm1 promoter, increased H3K27me3, decreased H3K4me3, and reduced Hotairm1 levels in a pattern similar to GSK-J4 treatment.

In summary, KDM6A demethylase activity is a potential therapeutic target in sepsis MDSCs, acting in part by driving Hotairm1 transcription. Hotairm1, in turn, modifies the vital inflammatory protein mediator, S100A9. Thus, epigenetic, post-translational, and nuclear protein mediators likely combine to inform MDSC development during later and possibly chronic sepsis with immunosuppression and rising mortality rates. Our findings support that Hotairm1 targeting is a likely treatment path for protracted sepsis.

## Data Availability Statement

The original contributions presented in the study are included in the article/**Supplementary Material**. Further inquiries can be directed to the corresponding author.

## Ethics Statement

The human study was approved by the Institutional Review Board (IRB) of the East Tennessee State University (IRB#: 0714.6s). All patients provided their written informed consent to participate in this study. All animal experiments were conducted in accordance with National Institutes of Health guidelines and were approved by the East Tennessee State University Animal Care and Use Committee (Protocol#: 190603).

## Author Contributions

IB conducted the experiments. DY recruited patients and collected blood samples. ZY provided critical input for experimental design and discussed the results. CM provided insights regarding data interpretation and edited the manuscript. MG designed the study, interpreted the data, and wrote the manuscript. All authors contributed to the article and approved the submitted version.

## Funding

This work was supported by a National Institutes of Health grant R35GM131692 (to MG). CM is supported by an NIGMS grant R35GM126922.

## Conflict of Interest

The authors declare that the research was conducted in the absence of any commercial or financial relationships that could be construed as a potential conflict of interest.

## Publisher’s Note

All claims expressed in this article are solely those of the authors and do not necessarily represent those of their affiliated organizations, or those of the publisher, the editors and the reviewers. Any product that may be evaluated in this article, or claim that may be made by its manufacturer, is not guaranteed or endorsed by the publisher.
