# Supplementary figures and images for: Inhibiting KDM6A Demethylase Represses Long Non-Coding RNA Hotairm1 Transcription in MDSC During Sepsis
Source: Front Immunol. 2022 Jan 28;13:823660. doi: 10.3389/fimmu.2022.823660 (PMC8851568; doi:10.3389/fimmu.2022.823660)

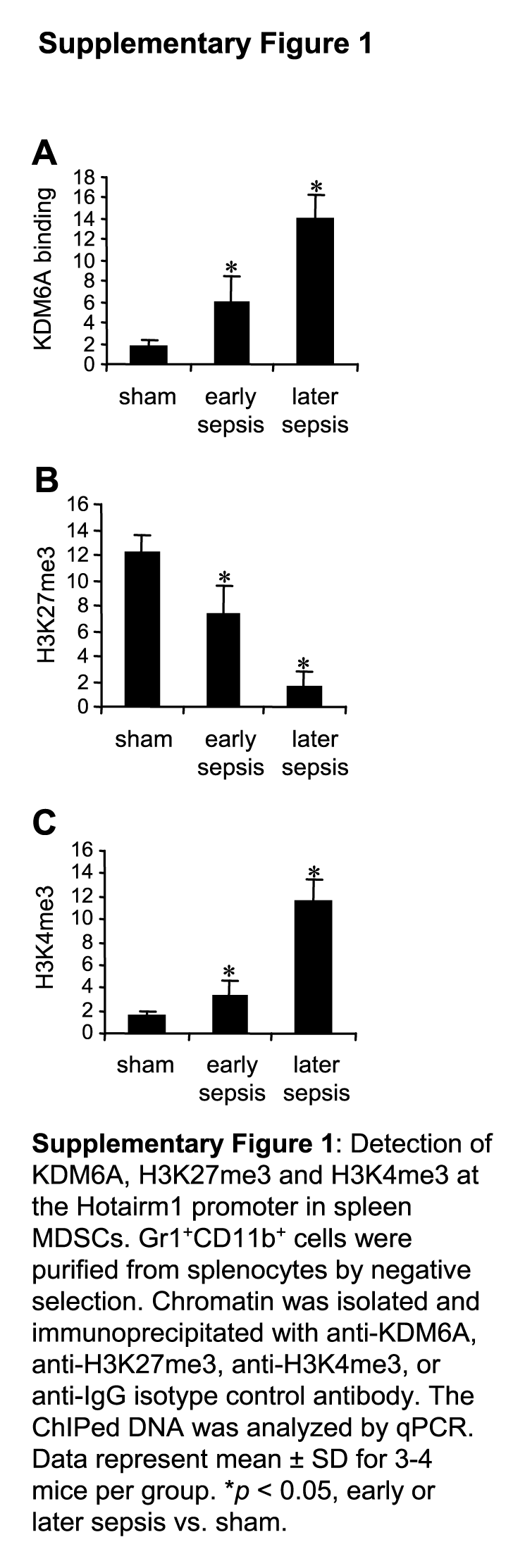

Supplement: Supplementary file 1 [file Image_1.tif]
