# Supplementary material for: Inhibiting KDM6A Demethylase Represses Long Non-Coding RNA Hotairm1 Transcription in MDSC During Sepsis
Source: Front Immunol. 2022 Jan 28;13:823660. doi: 10.3389/fimmu.2022.823660 (PMC8851568; doi:10.3389/fimmu.2022.823660)
Supplement: Supplementary file 2 [file Table_1.doc]

**Supplementary Table 1**. Antibodies and PCR primers used in this study.

| Cell analysis | antibody | source | catalog number |
| --- | --- | --- | --- |
|  | anti-CD11b-PE | eBioscience | 12-0112-82 |
|  | anti-Gr1-FITC | eBioscience | 11-5931-82 |
|  | anti-HLA-DR | eBioscience | 13-9956-82 |
|  | anti-CD33 | Invitrogen | MA1-19522 |
|  | anti-LOX-1 | Milteny Biotec | 130-122-119 |
| **ChIP** |  |  |  |
|  | anti-KDM6A | Cell Signaling Technology | 33510S |
|  | anti-Ezh2/ENX-1 | Cell Signaling Technology | 5246S |
|  | anti-PU.1 | Invitrogen | MA5-15064 |
|  | anti-H3K27me3 | MyBioSource | MBS3010330 |
|  | anti-H3K4me3 | MyBioSource | MBS9401981 |
| **Western blot** |  |  |  |
|  | anti-KDM6A | Cell Signaling Technology | 33510S |
|  | anti-Ezh2/ENX-1 | Cell Signaling Technology | 5246S |
|  | anti-PU.1 | Invitrogen | MA5-15064 |
|  | anti-IL-10 | Santa Cruz Biotechnology | sc-32815 |
|  | anti-S100A9 | Santa Cruz Biotechnology | sc-58706 |
|  |  |  |  |
| **ChIP PCR primers** | **forward** | **reverese** | **amplicon** |
| Mouse Hotairm1 promoter | 5'-tcccagagtcgccactgccaa-3' | 5'-tagagtcacgtgtcctcccc-3' | 220 bp |
|  |  |  |  |
| Human Hotairm1 promoter | 5'-gtatggggtattccaggaagg-3' | 5'-gaggctcagccattggctga-3' | 220 bp |
|  |  |  |  |
